# The Rarest of the Rare: A Case of BAP1-Mutated Primary Peritoneal Mesothelioma

**DOI:** 10.7759/cureus.18380

**Published:** 2021-09-29

**Authors:** Aanchal Gupta, Alisa Vasileva, Sukesh Manthri

**Affiliations:** 1 Internal Medicine, St. Martinus University Faculty of Medicine, Willemstad, CUW; 2 Research, Ballad Health, Johnson City, USA; 3 Oncology, Mary Bird Perkins Cancer Center, Houma, USA

**Keywords:** genetic testing, asbestos, mesothelioma, malignant peritoneal mesothelioma, bap1

## Abstract

Malignant mesotheliomas (MM), as described are rare tumors that are mostly associated with occupational exposure to asbestos. They most commonly occur in the pleura. Other unfamiliar sites where they can occur are the peritoneum, pericardium, and tunica vaginalis. There is no significant correlation between the amount and duration of asbestos exposure to mesothelioma development as reported by various studies over the years. Apart from the environmental exposure, the development of malignant mesothelioma has been linked to a mutation in the BAP1 gene, which can predispose the patient to develop other malignancies associated with BAP1 mutation. We report a case of a 43-year-old man without any significant risk factors, who presented with a complaint of abdominal discomfort and was found to have malignant peritoneal mesothelioma (MPM). With a known familial history of mesothelioma and melanoma, our patient underwent genetic testing which revealed a mutation in BAP1, affirming the strong association with the development of MPM. Young patients who develop malignant mesothelioma without risk factors for MM should have germline testing for BAP1. This case report is unique and highlights a familial variant of mesothelioma, even rare with peritoneal mesothelioma in our patient.

## Introduction

Malignant mesothelioma (MM) is a fairly uncommon cancer and is most commonly associated with exposure to asbestos. Roughly 3,000 cases of MM occur annually in the United States [1]. Mesothelial cells are known to line the pleura, pericardial and peritoneal cavities but the most common locations for mesothelioma development are visceral pleura followed by peritoneal cavities. It occurs predominantly in elderly men, 65 years or older. The most common mechanism by which asbestos causes MM development is through cytotoxicity induced by the asbestos fibers which lead to cell necrosis and release of high mobility group box protein-1 (HMGB1) which has been deduced as a cause for the initiation of asbestos-induced mesothelioma formation.

Besides asbestos, about 20-50% of individuals that develop MM have no history of asbestos exposure which indicates that there are other factors that cause mesothelioma or that some individuals are highly susceptible to even the slightest amounts of asbestos [2]. Novel studies have shown that germline alterations in BAP1 and homologous recombination (HR) DNA repair or tumor suppressors genes were most prevalent in individuals with MM [3]. Biallelic inactivation of BAP1, a deubiquitinase enzyme located on chromosomal region 3p21.2, was observed in familial mesotheliomas [4]. BAP1 has been shown to have various roles including cell cycle progression, DNA damage response/repair, and genomic instability in regards to MM tumorigenesis [1]. Recently, numerous studies have also talked about BAP1- associated cancer syndrome characterized by malignant mesothelioma, uveal melanoma, benign atypical melanocytic lesions, and other malignancies [5] which inherits in an autosomal dominant fashion.

Malignant peritoneal mesothelioma (MPM) is not diagnosed frequently at early stages due to a very vague presentation of symptoms ranging from abdominal distention to diffuse, nonspecific abdominal pain which is most often the cause of delayed diagnosis [6]. Studies have reported that 90% of mesotheliomas occur in the pleura whereas 2-7% occur in the peritoneum [7]. MPM occurs more commonly in females of younger age [8]. MPM is known to have shorter median survival than malignant pleural mesothelioma with a median survival of less than 1 year vs 4-12 for the latter [9]. This case report is unique and highlights a familial variant of mesothelioma, even rare with peritoneal mesothelioma in our patient.

## Case presentation

**Figure 1:**
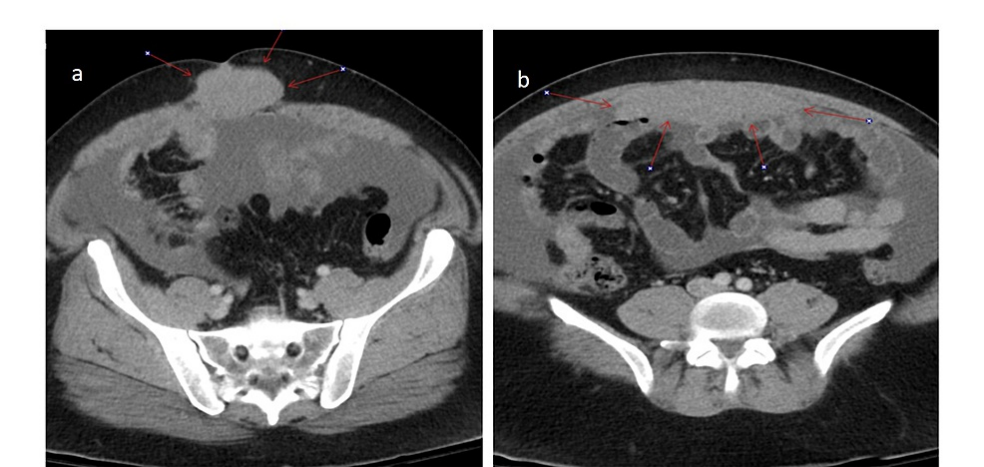
(a) Axial section of CT abdomen/pelvis with contrast showing mass in the umbilical region (arrows); (b) Axial section of CT abdomen/pelvis with contrast showing showed diffuse thickening and soft tissue attenuation of the omentum along with moderate volume ascites concerning for omental carcinomatosis (arrows).

**Figure 2:**
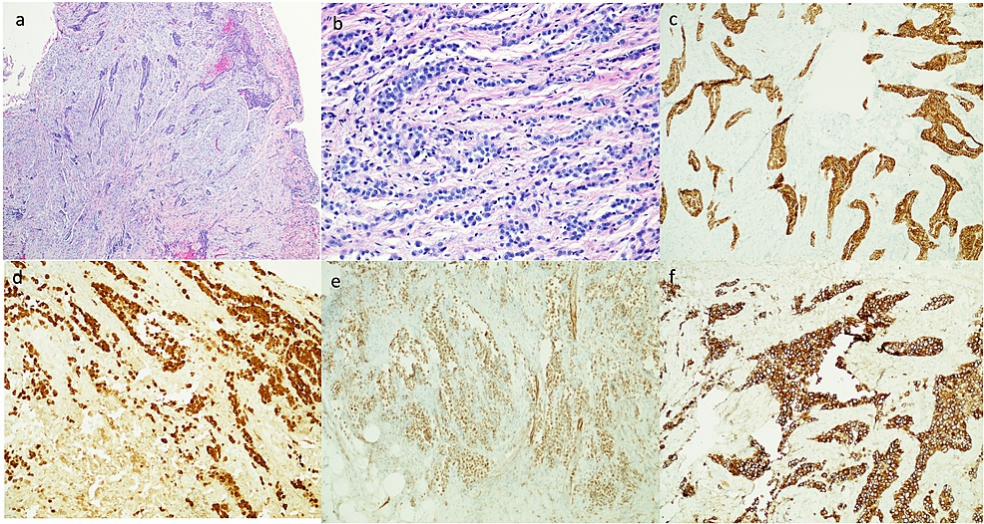
Histologic sections of the current omental and peritoneal biopsies demonstrate an infiltrative and tumefactive mesothelial proliferation composed of plump epithelioid cells with cytologic atypia (a & b). The tumor cells are positive for CK5/6 (c), calreticulin (d), mesothelin (e), and WT1 (f), confirming mesothelial origin. CK: cytokeratin; WT: Wilms' tumor

A 43-year-old man with a known history of sclerosing mesenteritis, a family history of mesothelioma, melanoma, and thyroid cancers presented with abdominal discomfort. CT abdomen/pelvis with contrast showed diffuse thickening and soft tissue attenuation of the omentum along with moderate volume ascites concerning for omental carcinomatosis (Figure 1 a & b) status post omental and peritoneal biopsies. Histologic sections of the current omental and peritoneal biopsies demonstrate an infiltrative and tumefactive mesothelial proliferation composed of plump epithelioid cells with cytologic atypia. The tumor cells are positive for cytokeratin (CK) 7, CK5/6, calreticulin, mesothelin, and Wilms tumor 1 (WT1), confirming mesothelial origin. Biopsy was consistent with malignant mesothelioma, epithelioid type (Figure 2). Solid tumor genomic assay showed no somatic mutations, stable MSI (microsatellite instability), no amplification, or gene fusion. With a known maternal family history of mesothelioma, genetic testing revealed the presence of a pathogenic mutation in the BAP1 gene, described as c.2050C>T.

The patient was evaluated at tertiary care high volume center. At that time it was unclear if he will be a candidate for cytoreductive surgery and HIPEC (hyperthermic intraperitoneal chemotherapy). He has received cisplatin 75 milligram/meter sq and pemetrexed 500 milligram/meter sq every 21 days and due to poor tolerance cisplatin was changed to carboplatin eventually. CT chest/abdomen/pelvis done after 2 months has shown response to chemotherapy however done at 4-month interval has shown enlarging mediastinal and bilateral supraclavicular lymphadenopathy, an increase in the volume of ascites, and a slight decrease in peritoneal carcinomatosis. He was started on second-line nivolumab 360 mg and ipilimumab 1 milligram/kg. CT chest/abdomen/pelvis with contrast 11 months after initiation of immunotherapy has shown interval increase in the size of the thoracic nodes, increase disease volume along the perigastric region and gastrohepatic region concerning for disease progression. Was started on vinorelbine 30 milligram/meter sq in the 3rd line setting. The patient had significant challenges obtaining central line access (port placement, PICC line) due to extensive mediastinal adenopathy leading to treatment daily. Status post three cycles of chemotherapy and scans show stable disease. 

## Discussion

Malignant mesothelioma is a rare disease predominantly caused by asbestos exposure but has also been linked to genetic predisposition due to somatic mutations in certain genes. Mesothelioma arises from the mesothelial cells lining the pleura, peritoneum, and very rarely from the pericardial cavity and tunica vaginalis. It occurs most commonly in the pleura but 10-30% of all mesotheliomas originate in the peritoneum [10]. Malignant peritoneal mesothelioma is more frequent in younger females compared to older males in pleural mesothelioma and has a better prognosis in contrast to sporadic mesotheliomas [11], making our case report unique, as we have a young male with a family history of mesothelioma.

The pathogenesis of mesothelioma is attributed to chronic inflammation caused by asbestos exposure which causes cell necrosis and cells that do survive after the inflammation, may undergo mutations causing the formation of mesothelioma over a period of years. Asbestos exposure not only occurs in occupational settings, but home renovations are also one of the leading causes of non-occupational setting exposure [7]. MPM has a weaker link with asbestos exposure indicating other factors responsible for its development. Mutation of the tumor suppressor gene, BAP1, responsible for the suppression of BRCA** **(breast cancer gene)-mediated cell growth has been linked to the high incidence of many malignancies, including mesothelioma and uveal melanoma. BAP1 cancer syndrome is known to be transmitted in Mendelian fashion, making it highly penetrant [12]. BAP1 is known to localize in the nucleus and cytoplasm where it performs different functions, specifically, regulates DNA repair in the nucleus and aerobic respiration and programmed cell death/apoptosis in the cytoplasm. Therefore, due to these mechanisms, people with BAP1 mutation carry a high risk for cancer, especially those cancers which are caused by environmental carcinogens [12].

Data indicates that only 1% of mesotheliomas can be attributed to germline BAP1 mutation [13]. People who carry BAP1 mutation have a higher ratio of peritoneal to pleural mesothelioma compared to the rate of occurrence of sporadic mesotheliomas [14]. Thus, making genetic testing very important for early detection of any malignancies associated with BAP1 cancer syndrome. Our patient also underwent genetic testing, which showed a BAP1 mutation inheritance in the family.

Mutations in BAP1 have been associated with hereditary predisposition to uveal melanoma, cutaneous melanoma, renal cell carcinoma, mesothelioma, and other tumors. This condition is relatively newly described (clinical phenotype described in the literature [4]), and the full spectrum of cancer in tumor risk has not been entirely established at this time. The mutation identified likely explains the diagnosis of peritoneal mesothelioma and his family history of mesothelioma and skin cancers including melanoma. At this time, there are no consensus guidelines on the screening or cancer management for individuals identified to have BAP1 germline mutation.

A recent publication by Rai et al [15] suggests the following screening measures for individuals with a germline BAP1 mutation: - Uveal melanoma: Annual dilated eye exams and off thymic imaging by ocular oncologist beginning at age 11. If uveal melanoma is identified, consider high-risk monitoring for metastatic surveillance, including liver-directed imaging every 3-6 months, pulmonary imaging every 6-12 months. - Malignant mesothelioma: Yearly physical examinations - Cutaneous melanoma, basal cell carcinoma, atypical spitz tumors: Yearly dermatological full-body skin exam beginning at age 20, self-skin exam, use of sun protection. - Renal cell carcinoma: Can consider the VHL (Von-Hippel Lindau) screening protocol (annual abdominal ultrasound exam, MRI every 2 years).

First-degree family members have a 50% likelihood of also carrying the BAP1 mutation. Printed genetic testing would be indicated for at-risk family members. Our patient's children have undergone genetic testing and one of the two kids was positive for the familial BAP1 mutation. The symptoms associated with malignant peritoneal mesothelioma are nonspecific. Ascites and abdominal discomfort/ pain are among the most common complaints. The most common imaging modalities are CT and MRI for detection of MPM which usually reveals peritoneal and mesenteric thickening [16]. A biopsy is very important for establishing the diagnosis which can be done either radiographically or surgically. Our patient is a 43-year-old male, 39 when diagnosed, who presented with abdominal discomfort and had a family history of mesothelioma. After undergoing the CT and biopsy, it was revealed that our patient had epithelioid type which is the most common type representing 75-90% of cases, besides, sarcomatoid and biphasic and is known to have a better prognosis. MPM is shown to be more expansive than infiltrative, in contrast to our patient who had an infiltrative etiology.

The treatment options for malignant peritoneal mesothelioma were very limited. If operable stages, the new standard of care is CRS-HIPEC (cytoreductive surgery and hyperthermic intraperitoneal chemotherapy) which has shown to be the mainstay treatment option along with the systemic therapies. Unfortunately, our patient was not eligible for CRS-HIPEC and was started on palliative systemic chemotherapy. Pemetrexed-based regimens are the first-line systemic chemotherapy instituted at most places [6]. It is usually used along with cisplatin/carboplatin depending on the tolerability of the patient but carboplatin has shown to be tolerated better with the same efficacy as cisplatin [6]. The patient in this study did respond to the pemetrexed/carboplatin therapy initially but after 4 months, we had to switch to the second-line treatment due to disease progression. The role of immunotherapy in peritoneal mesothelioma is evolving. Even though there is no established second-line treatment, the recent advancement in immune checkpoint inhibitors has shown some promising results in the treatment of MPM. Nivolumab and ipilimumab are PD1 [programmed cell death protein-1) and CTL4 (cytotoxic T-lymphocyte-associated protein-4) checkpoint inhibitors, which when combined together, induce synergistic responses as shown in MAPS2 and INITIATE trials [16, 17]. Monotherapy with either agent has shown very low efficacy in MPM as compared to other malignancies [18]. The patient in our report received 11 months of combination therapy of nivolumab and ipilimumab before disease progression. Our patient was started on vinorelbine in a third-line setting, although the efficacy and survival rate of vinorelbine is unclear and questionable [19, 20]. 

## Conclusions

Malignant mesothelioma and BAP1 associated cancer syndromes occur at a very young age. In people with strong family history, it is imperative to do genetic testing for BAP1 mutation. Early detection of this mutation has shown to increase the overall survival in these predisposed people due to close monitoring, more therapeutic options for early stages of cancer, and implementing preventative measures to limit the environmental carcinogenic exposures.
